# Assessing Ultrasound as a Tool for Monitoring Tumor Regression During Chemotherapy: Insights from a Cohort of Breast Cancer Patients

**DOI:** 10.3390/cancers17101626

**Published:** 2025-05-11

**Authors:** Vlad Bogdan Varzaru, Aurica Elisabeta Moatar, Roxana Popescu, Daniela Puscasiu, Daliborca Cristina Vlad, Cristian Sebastian Vlad, Andreas Rempen, Ionut Marcel Cobec

**Affiliations:** 1 Doctoral School, Faculty of Medicine, “Victor Babes” University of Medicine and Pharmacy Timisoara, 300041 Timisoara, Romania; 2 ANAPATMOL Research Center, Faculty of Medicine, “Victor Babes” University of Medicine and Pharmacy Timisoara, 300041 Timisoara, Romania; 3 Clinic of Obstetrics and Gynecology, Diakoneo Diak Klinikum, 74523 Schwäbisch Hall, Germany; 4 Clinic of Internal Medicine-Cardiology, Klinikum Freudenstadt, 72250 Freudenstadt, Germany; 5 Department of Cell and Molecular Biology, “Victor Babes” University of Medicine and Pharmacy Timisoara, 300041 Timisoara, Romania; 6 Department of Pharmacology, “Victor Babes” University of Medicine and Pharmacy Timisoara, 300041 Timisoara, Romania; 7 Clinic of Obstetrics and Gynecology, Klinikum Freudenstadt, 72250 Freudenstadt, Germany

**Keywords:** breast cancer, ultrasound, chemotherapy

## Abstract

Background/Objectives: Accurate assessment of tumor response to neoadjuvant chemotherapy (NAC) in breast cancer is critical for optimizing treatment strategies. While magnetic resonance imaging (MRI) and mammography are commonly used for response evaluation, they have inherent limitations. Ultrasound (US) has emerged as a promising, cost-effective, and real-time alternative. This study aimed to evaluate the effectiveness of US in tracking tumor regression during NAC and its correlation with pathologic tumor regression grade (TRG). Methods: This study included 282 breast cancer patients undergoing NAC. Tumor size was measured using ultrasound at three key time points: pre-chemotherapy, after four cycles, and post-chemotherapy. Spearman’s correlation was used to assess the relationship between US-measured tumor changes and TRG. Multinomial logistic regression and receiver operating characteristic (ROC) curve analyses were performed to determine the predictive accuracy of the measurements from our US in identifying pathologic complete response (pCR). Conclusions: Ultrasound is a reliable, real-time imaging tool for monitoring NAC response in breast cancer patients. Its ability to predict pCR and track tumor shrinkage highlights its potential for treatment adaptation. Standardization of US protocols and integration with AI-based analysis may further improve its clinical utility, making it a valuable adjunct in breast cancer treatment monitoring.

## Simple Summary

Breast cancer remains a significant health concern worldwide, with chemotherapy playing a crucial role in treatment. Monitoring tumor response to therapy is essential for optimizing treatment decisions and improving patient outcomes. Traditional imaging methods like MRI and mammography are widely used but have limitations, such as cost, availability, and accuracy. Ultrasound offers a cost-effective, real-time, and non-invasive alternative for tracking tumor regression during chemotherapy. Our study evaluated the effectiveness of ultrasound in assessing tumor shrinkage and its correlation with pathological response. By comparing ultrasound measurements at different chemotherapy stages, we aimed to determine its predictive accuracy in identifying patients who may benefit from treatment adjustments. Our findings could contribute to improving breast cancer management by integrating ultrasound as a standard tool for treatment monitoring, ultimately leading to more personalized and effective care.

## 1. Introduction

Breast cancer remains a significant global health challenge, with its incidence continuing to rise worldwide. Effective monitoring of tumor response to chemotherapy is crucial for optimizing treatment strategies and improving patient outcomes [1]. Among breast cancer subtypes, triple-negative breast cancer (TNBC) presents unique challenges, due to its aggressive nature, higher recurrence rates, and limited targeted treatment options [2]. Neoadjuvant chemotherapy (NAC) is commonly used to reduce tumor size, facilitate breast-conserving surgery, and assess tumor responsiveness to systemic therapy. Traditionally, pathologic tumor size (pT) has been considered the gold standard for evaluating tumor regression following chemotherapy. However, real-time, non-invasive monitoring tools are increasingly necessary to personalize treatment decisions and predict therapeutic outcomes [3]. Emerging evidence suggests that, beyond standard imaging modalities, ultrasound-based quantitative imaging techniques, including radiomics and artificial-intelligence-driven models, may enhance the assessment of tumor response and provide a more personalized approach to treatment monitoring [4]. Moreover, advancements in ultrasound texture analysis and tumor echogenicity assessments have shown potential in differentiating chemotherapy responders from non-responders, providing valuable real-time treatment insights [5].

Among the various imaging modalities, magnetic resonance imaging (MRI) and mammography have been widely used to assess tumor response to chemotherapy. However, these techniques have limitations. Mammography often overestimates tumor dimensions, leading to potential overtreatment, while MRI, though highly sensitive, is costly and not always readily available [6,7]. US has emerged as a promising, cost-effective, and dynamic alternative for tracking tumor regression over time [8]. US allows for real-time tumor monitoring, is more accessible than MRI, and provides detailed anatomical and structural insights into tumor changes during chemotherapy.

Recent studies have investigated the role of ultrasound in monitoring tumor response. Piotrzkowska-Wróblewska et al. evaluated tumor response to NAC by analyzing backscattered ultrasound energy and backscatter statistics. Their findings suggested that ultrasound imaging, when integrated with statistical analysis, can enhance the personalization of NAC regimens, improving patient outcomes [9]. Additionally, advanced machine learning models, such as deep-learning-based image analysis, have been explored for predicting chemotherapy response from imaging data, particularly in HER2-positive breast cancer patients, offering promising avenues for individualized treatment planning [10]. Furthermore, novel ultrasound-based texture analysis methods have demonstrated the ability to assess tumor microstructural changes, which may serve as an early indicator of therapy response, potentially improving patient stratification and treatment adaptation [5,11].

Despite these advances, significant research gaps remain regarding the predictive value of ultrasound. While mammography and MRI have well-established predictive capabilities, ultrasound remains underutilized as a quantitative tool for longitudinal assessment of tumor regression. Notably, some studies suggest that tumor volume changes measured via ultrasound correlate inconsistently with pathologic complete response (pCR), highlighting the need for further validation of US as a predictive tool [12]. Additionally, there has been limited research exploring the relationship between ultrasound-derived tumor regression metrics and final pathological outcomes at different stages of chemotherapy [13]. The incorporation of quantitative ultrasound parameters, such as echogenicity changes and textural pattern shifts, may help refine tumor response assessment and further validate its role as a reliable tool in monitoring treatment efficacy [4,5].

This study aimed to evaluate the ability of ultrasound to monitor tumor regression at three key chemotherapy time points: pre-chemotherapy, mid-treatment, post-chemotherapy. We assessed the correlation between ultrasound-measured tumor changes and pathologic tumor regression grade (TRG). TRG is a histopathological indicator that quantifies the extent of tumor cell reduction and fibrosis after NAC and is associated with patient prognosis [14]. Studies in rectal and gastric cancer have demonstrated a strong prognostic role for TRG, yet its correlation with ultrasound-based tumor size reductions in breast cancer remains unclear [15].

By establishing this correlation, we hypothesize that ultrasound can serve as a reliable, real-time, and non-invasive surrogate for pT, facilitating early treatment response assessment and potentially guiding modifications to chemotherapy regimens. The findings of this study could help bridge the existing knowledge gaps, supporting the integration of ultrasound into routine oncological practice for monitoring tumor response to therapy.

## 2. Materials and Methods

Our study analyzed retrospective anonymized breast cancer data of patients who received chemotherapy and where tumor monitoring was performed with ultrasound. The anonymized breast cancer data were recorded between January 2010 and December 2021, at the Clinic of Obstetrics and Gynecology, Diakoneo Diak Klinikum Schwäbisch Hall, Germany.

In this study, we conducted a comprehensive statistical analysis to evaluate the performance of ultrasound in monitoring tumor regression during chemotherapy and to compare its efficacy to pathologic tumor size. The analysis was structured to provide insights into the predictive value of the measurements performed with our US at different stages of treatment and to understand their relationship with clinical outcomes.

We began by summarizing continuous variables using medians and interquartile ranges, due to their non-normal distributions, confirmed by a Shapiro–Wilk test. Categorical variables were described using counts and proportions, to illustrate the distribution of key clinical and demographic characteristics.

Spearman’s rank correlation was performed to assess the relationship between TRG and the measurements carried out at each stage with our US (pre-chemotherapy, after four cycles, and post-chemotherapy), as well as the percentage change from pre- to post-chemotherapy. This non-parametric test was chosen due to the non-normal distribution of the data, and it allowed us to evaluate the strength and direction of associations.

A Kruskal–Wallis test was used for comparing US measurements and percentage changes across different pT categories and hormone receptor statuses. This non-parametric method was selected due to the data’s non-normal distribution, and post hoc Dunn-Bonferroni tests were applied for pairwise comparisons where significant differences were detected.

To assess tumor size changes over time, we conducted a Friedman test to compare the measurements carried out at the three time points (before chemotherapy, after four cycles, and after chemotherapy). This test, suitable for repeated measures within subjects, was followed by a generalized estimating equations (GEE) model, which accounted for patient ID as a random effect, to model the longitudinal data while adjusting for within-subject correlations.

We developed three multinomial logistic regression models, using US measurements before chemotherapy, after four cycles, and after chemotherapy as predictors, with pT as the response variable. This analysis aimed to determine the predictive value of US at each stage for different pT outcomes. Only significant predictors were retained in the final models, and model performance was assessed using Nagelkerke’s R^2^ to gauge the explanatory power.

A threshold of ≥30% reduction in tumor size was used to define significant regression, consistent with RECIST 1.1 criteria for partial response [16]

To evaluate and compare the performance of the US models at different stages, we computed the area under the curve (AUC) for each pT class. This step allowed us to assess the diagnostic accuracy of ultrasound measurements at each stage in identifying specific pathologic outcomes.

The ultrasound measurements were performed using the same ultrasound system across all patients. Although the specific model was not recorded, all examinations adhered to a standardized imaging protocol used routinely at our institution. The protocol included grayscale B-mode imaging for tumor size assessment, supplemented by Doppler ultrasound to evaluate vascularity. All measurements were conducted by a consistent team of experienced sonographers, ensuring procedural uniformity throughout the study. While formal intra- and inter-observer reproducibility testing was not performed retrospectively, all operators followed institutional quality assurance standards.

Pathologic tumor regression was assessed using the Sinn–Denkert system, which classifies tumor response into five grades based on residual tumor cellularity and fibrosis. This system has been widely validated and used in clinical trials to assess response to neoadjuvant chemotherapy.

Finally, we performed logistic regression to examine the odds of achieving significant tumor regression (defined as pT0 or pTis) based on the percentage change in sonographic measurements from pre- to post-chemotherapy. Predictive accuracy was visualized using an ROC curve, with sensitivity, specificity, and AUC reported to illustrate the discriminatory power of the sonographic measurement changes in comparison to the gold standard, pT.

All analyses were conducted using a significance threshold of *p* < 0.05, and results were reported with 95% confidence intervals where applicable. All statistical analyses were conducted using R (version 4.3.0; R Core Team, 2023) and RStudio (version 2023.06.0+421; RStudio Team, 2023), ensuring robust and reproducible findings.

## 3. Results

In this study, we utilized a comprehensive set of variables to analyze tumor response to chemotherapy in a cohort of 282 female breast cancer patients. Ultrasound measurements of tumor size were taken at three key time points: before chemotherapy, after four cycles of chemotherapy, and post-chemotherapy. These measurements were used to derive percentage change variables that quantified the reduction in tumor size over the course of treatment.

Clinical and biological variables included TRG, which assessed the degree of pathologic response post-chemotherapy, and pathologic tumor size (pT), serving as the gold standard for evaluating treatment success. Hormone receptor statuses—estrogen receptor (ER), progesterone receptor (PR), and HER-2/neu—provided insight into tumor subtypes and their potential influence on treatment response.

The majority of tumors were estrogen receptor (ER)-positive (57.8%), with 46.8% also positive for progesterone receptor (PR). HER2 positivity was observed in 37.2% of cases. Most patients had no associated ductal carcinoma in situ (DCIS) (94%). Tumor regression grade (TRG) was distributed across the cohort, with TRG 1 (34%) and TRG 4 (41.1%) being the most common. Pathologic tumor size (pT) after chemotherapy varied, with 41.5% achieving a complete response (pT0), while the remainder were distributed primarily among T1 and T2 categories. The tumors were nearly evenly distributed between left (53.9%) and right (46.1%) breasts. The results are presented in Table 1.

The study also accounted for whether patients received neoadjuvant chemotherapy and included the Ki67 proliferation index, a marker indicative of tumor aggressiveness.

The ultrasound measurements for tumor size before, during, and after chemotherapy demonstrated significant variability, as seen through the interquartile ranges (IQR) and median values. Initially, the median ultrasound-measured tumor size before chemotherapy was 27 mm, with an interquartile range of 19 to 36 mm, reflecting the typical size distribution among patients at baseline. After four cycles of chemotherapy, this measurement decreased substantially, with a median of 16 mm (IQR: 11–23 mm), suggesting a notable reduction in tumor size in response to treatment by this intermediate stage. By the end of chemotherapy, the median ultrasound-measured tumor size had further declined to 12 mm (IQR: 8–18 mm), indicating continued tumor regression throughout the course of therapy. This progressive decrease across all three time points suggests that ultrasound can effectively capture the dynamics of tumor shrinkage, aligning with the expected therapeutic impact of chemotherapy over time.

The Shapiro–Wilk test results for ultrasound measurements across all three time points yielded *p*-values of less than 0.001, indicating significant departures from normality. This non-normal distribution underscores the heterogeneous response of tumors to chemotherapy in this cohort, which may have been due to biological variability in tumor biology, individual patient response to chemotherapy, or other clinical factors. The non-parametric nature of these distributions highlights the importance of considering individual variability when interpreting tumor size changes, as well as the potential value of ultrasound for monitoring individual patient responses.

Additionally, Ki67, a commonly used marker of cellular proliferation, displayed a median value of 0.30 (IQR: 0.20–0.50), with a Shapiro–Wilk *p*-value of less than 0.001, also indicating a non-normal distribution. This finding suggests considerable variability in proliferative activity among the patients, which may correlate with tumor aggression and response to chemotherapy. The non-normal distribution of Ki67 values further reinforces the heterogeneity within this patient cohort, potentially impacting both the speed and degree of tumor regression observed on ultrasound. The results are presented in Table 2.

### 3.1. Analysis of Percentage Change in Tumor Size Across Chemotherapy Stages

To quantify changes in tumor size at key stages of chemotherapy, we derived percentage change variables based on ultrasound measurements taken at three critical time points: before chemotherapy, after four cycles of chemotherapy, and post-chemotherapy. Specifically, we calculated the percentage change from the initial measurement before chemotherapy to the measurement after four cycles, the change from the four-cycle measurement to the final post-chemotherapy measurement, and the cumulative change from pre-chemotherapy to post-chemotherapy. Each of these variables represented the proportionate reduction in tumor size relative to the initial size at each stage, providing a standardized view of how much the tumor had shrunk by each interval.

The pre-chemotherapy to four-cycle change variable reflected the early response to treatment, showing the initial impact of chemotherapy on tumor reduction. The four-cycle to post-chemotherapy change variable then captured the additional reduction achieved in the later phase of treatment. Lastly, the pre-chemotherapy to post-chemotherapy change variable offered a complete view of the overall reduction in tumor size across the entire course of chemotherapy, highlighting the cumulative impact of the treatment regimen.

Our analysis showed that these percentage changes differed significantly across the stages. The median reduction from pre-chemotherapy to the end of chemotherapy was 52.44%, indicating a strong overall response. The initial change from pre-chemotherapy to four cycles was 36.24%, demonstrating that a substantial portion of tumor shrinkage occurred early in treatment. This was followed by a smaller yet statistically significant additional reduction of 22.22% from the four-cycle to post-chemotherapy stage, suggesting a deceleration in response as treatment progressed.

**Figure 1:**
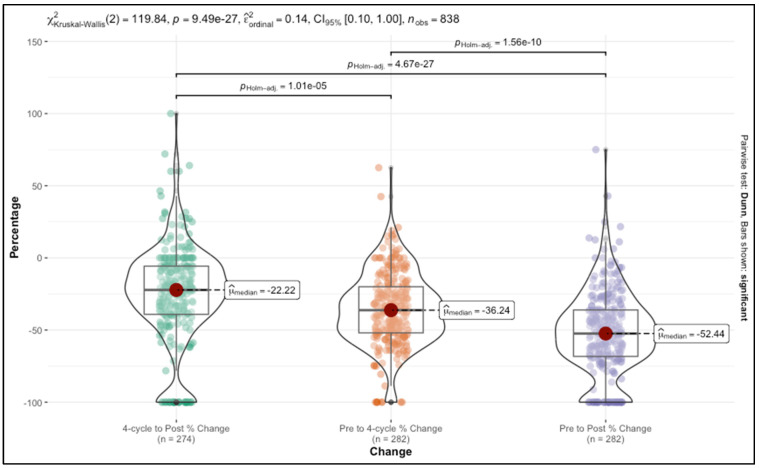
Distribution of percentage changes in tumor size across chemotherapy stages.

The statistical significance of these differences was confirmed by a Kruskal–Wallis test, with a chi-square value of 119.84 and a *p*-value less than 9.49 × 10^−27^. This strong result indicates that the tumor reduction percentages were not only clinically meaningful but also statistically distinct across the different chemotherapy stages. Post hoc comparisons showed that each stage differed significantly from the others, reinforcing the notion that tumor response evolves in progressive phases throughout chemotherapy. The results are presented in Figure 1.

### 3.2. Evaluating the Relationship Between Ultrasound Measurements and Tumor Regression Grade

The correlation analysis between tumor regression grade (TRG) and ultrasound measurements provided valuable insight into how effectively these imaging assessments reflected the true tumor response to chemotherapy. Across the entire cohort, the analysis demonstrated statistically significant negative correlations between TRG and ultrasound measurements at all time points, as well as with the percentage change in tumor size from pre-chemotherapy to post-chemotherapy. Specifically, TRG correlated with US before chemotherapy with a Rho of −0.248 (*p* < 0.001), US after four cycles with a Rho of −0.448 (*p* < 0.001), and US after chemotherapy with a Rho of −0.547 (*p* < 0.001). The correlation with the percentage change from pre-chemotherapy to post-chemotherapy was −0.488 (*p* < 0.001). These results suggest that higher TRG (indicating greater tumor regression) is associated with lower ultrasound-measured tumor sizes and more substantial percentage changes over the treatment course, affirming the utility of ultrasound in reflecting pathologic response. The importance of correlating TRG with percentage change from pre-chemotherapy to post-chemotherapy lies in its ability to capture the entire course of tumor response. This cumulative metric is crucial, as it represents the total impact of the chemotherapy regimen, providing a comprehensive view that single-point measurements cannot fully convey. The strong negative correlation here supports the use of ultrasound-derived changes as a proxy for pathologic assessment, enabling real-time tracking of therapeutic effectiveness. The results are presented in Table 3.

### 3.3. Longitudinal Analysis of Tumor Response to Chemotherapy Using Ultrasound Measurements

**Figure 2:**
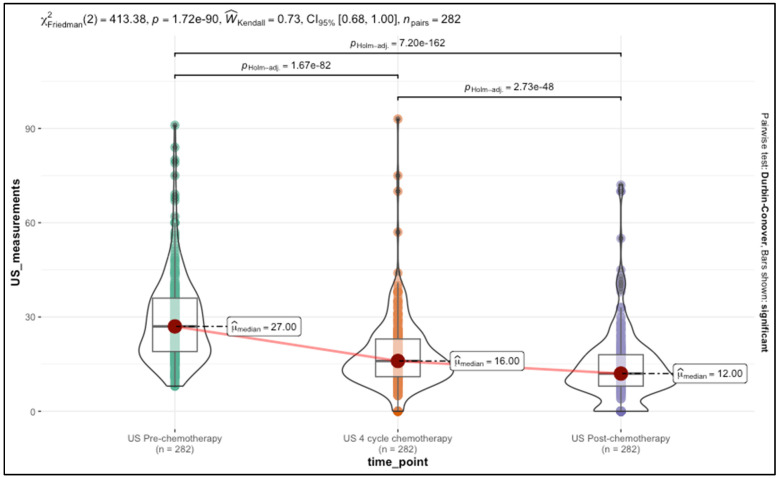
Distribution of ultrasound measurements across chemotherapy time points.

The longitudinal analysis was a crucial step in our study to assess tumor response over time and ensure that individual patient variability was considered. By performing a Friedman test, we first established that significant differences existed in the ultrasound measurements taken at different time points—before chemotherapy, after four cycles, and post-chemotherapy. The test results (χ^2^ = 413.28, *p* < 7.29 × 10^−90^) demonstrated strong statistical evidence that the median ultrasound measurements varied across these time points, indicating that the progression of tumor regression was not uniform throughout the treatment period. The results are presented in Figure 2.

To further quantify these changes, while accounting for repeated measures within the same patients, we implemented a generalized estimating equations (GEE) model. This approach was essential for incorporating the dependency between measurements taken from the same individual over time, which strengthened the robustness of our findings and allowed us to analyze the longitudinal trajectory of tumor response across the cohort while considering patient-specific effects.

The GEE model yielded significant results for both time points and other predictors. The time point of ultrasound after four cycles of chemotherapy showed an estimated reduction of −10.69 mm (CI: −11.86 to −9.53, *p* < 0.001) compared to the baseline, while post-chemotherapy measurements reflected an even greater reduction of −14.88 mm (CI: −16.19 to −13.58, *p* < 0.001). These findings indicate a progressive decrease in tumor size, with the greatest shrinkage observed by the end of chemotherapy. This supports the use of ultrasound for ongoing monitoring, showing that the treatment had a cumulative effect, with significant tumor size reduction as chemotherapy progressed.

The regression grade was a significant predictor, with an estimate of −3.15 mm (CI: −3.98 to −2.32, *p* < 0.001), confirming its strong negative association with tumor size at all time points. This means that higher regression grades (indicating greater tumor regression) were consistently associated with smaller tumor sizes, reinforcing the reliability of ultrasound in tracking true pathologic response over time.

HER-2/neu positivity was also significant, with an estimate of −3.09 mm (CI: −5.12 to −1.05, *p* = 0.003). This suggests that HER-2 positive patients had generally smaller tumor sizes during the treatment period, aligning with the known responsiveness of HER-2 positive tumors to the targeted therapies often administered alongside chemotherapy. The McFadden R^2^ value of 0.090, while modest, reflects the complexity of tumor response, which can be influenced by numerous biological and treatment-related factors. The results are presented in Table 4.

### 3.4. Ultrasound-Detected Tumor Size Reduction by Hormone Receptor and HER2 Status

To explore the relationship between molecular markers and ultrasound-assessed tumor response, we compared percentage tumor size reduction from pre- to post-chemotherapy across hormone receptor and HER2 status groups. Median tumor reduction was significantly greater in HER2-negative patients (−58.3%, IQR: 27.9%) compared to HER2-positive patients (−50.0%, IQR: 34.6%; *p* = 0.048).

Similarly, ER-positive patients demonstrated a higher median reduction (−59.1%, IQR: 31.2%) than ER-negative patients (−48.0%, IQR: 30.5%; *p* = 0.005). A comparable pattern was observed for PR status, with PR-positive patients showing a median reduction of −58.0% (IQR: 32.3%) versus −47.3% (IQR: 29.9%) in PR-negative patients (*p* = 0.004). The results are presented in Table 5.

### 3.5. Multinomial Logistic Regression Analysis of Ultrasound Predictive Power for Pathologic Outcomes

The analysis of multinomial logistic regression models using ultrasound measurements as predictors and pathologic tumor size (pT) categories as response variables was crucial for assessing the predictive power of US at different stages of chemotherapy. This analysis was designed to understand how well ultrasound measurements before treatment, after four cycles, and after the completion of chemotherapy could differentiate between various pT outcomes. By comparing the predictive capabilities of these models and evaluating their AUC (area under the curve) scores, we aimed to identify which stage of US measurement provided the most reliable information for predicting final pathologic outcomes.

The model using ultrasound measurements before chemotherapy showed significant associations across all pT categories. The odds ratios (OR) increased progressively with higher pathologic stages (e.g., OR = 1.26 for T3 and OR = 1.18 for T4b), indicating that larger initial tumor sizes were predictive of higher pathologic stages after chemotherapy. The Nagelkerke R^2^ value of 0.187 suggested that this model had modest explanatory power, implying that baseline ultrasound measurements alone provided some insight into future pT but were not highly predictive. The results are presented in Table 6.

The model using ultrasound measurements after four cycles showed stronger associations, particularly with higher pT categories. The OR for T3 was 1.48 (*p* < 0.001), and for T4b, it was 1.35 (*p* = 0.006), demonstrating that intermediate ultrasound measurements could better predict higher residual tumor burdens. The Nagelkerke R^2^ of 0.366 indicated an improvement in the explanatory power compared to the baseline model, reflecting that tumor measurements taken after initial treatment cycles were more informative for predicting pT outcomes. The results are presented in Table 7.

The post-chemotherapy model showed the highest predictive power, with significant odds ratios across various pT categories, such as OR = 1.41 for T2 (*p* < 0.001) and OR = 1.47 for T4b (*p* = 0.002). This model had the highest Nagelkerke R^2^ value of 0.505, indicating that final ultrasound measurements were the most effective in predicting the pathologic outcomes. The strong predictive power at this stage emphasizes the utility of ultrasound at the end of chemotherapy for assessing treatment effectiveness and planning subsequent clinical interventions. The results are presented in Table 8.

The final step of comparing the performance of ultrasound measurements to the gold standard (pathologic tumor size, pT) was essential to validate the effectiveness of US as a reliable tool for assessing significant tumor regression. This step provided critical evidence for the practical utility of US in clinical settings, allowing us to determine its accuracy in identifying true tumor response in comparison to the definitive pathologic outcome. By constructing this comparison, we could directly assess how well ultrasound measurements aligned with the final pathological assessments and determine the potential of US as a non-invasive surrogate for pT in evaluating treatment success.

To make this comparison, we created binary classification variables to indicate significant tumor regression according to both pathology and ultrasound assessments. Specifically, patients were categorized as having significant regression by pT if they achieved a status of T0 or Tis. For the measurements performed with our ultrasound device, we considered a regression significant if the percentage change in tumor size from pre- to post-chemotherapy was 30% or greater.

An ROC curve was then generated to assess the overall performance of the measurements in predicting significant tumor regression as defined by the gold standard (pT). The resulting plot and associated statistics provided a quantitative measure of the diagnostic power of US, comparing sensitivity and specificity to determine how well US could correctly identify cases of significant regression.

**Figure 3:**
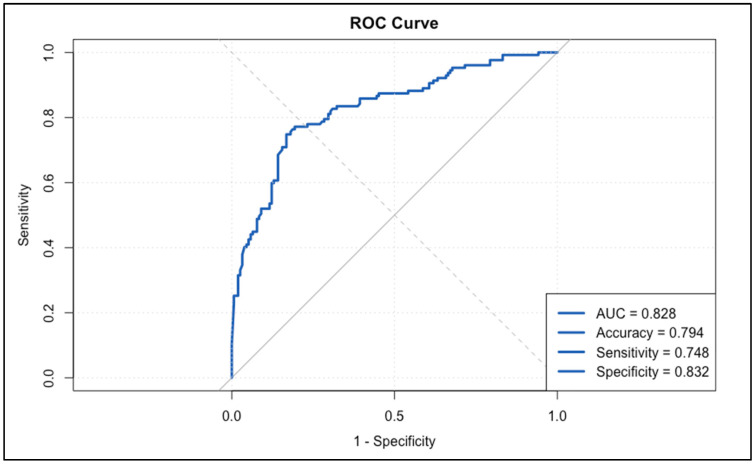
ROC curve for ultrasound performance in predicting significant tumor regression.

The ROC curve showed an AUC (area under the curve) of 0.828, which indicated a high level of accuracy in discriminating between patients who did and did not achieve significant tumor regression according to pT. An AUC value above 0.8 is generally considered strong, suggesting that US could reliably predict treatment success in a significant portion of cases. The associated accuracy of 0.794, sensitivity of 0.748, and specificity of 0.832 further supported this conclusion, demonstrating that the US measurements had a robust balance between true positive and true negative rates. These results validated the use of the 30% threshold in our cohort, confirming its discriminatory ability to distinguish between patients who achieved significant pathological regression (pT0 or pTis) and those who did not. The results are presented in Figure 3.

The logistic regression analysis reinforced these findings by showing that the percentage change from pre- to post-chemotherapy was a significant predictor of achieving significant regression (OR = 0.96, *p* < 0.001). This means that greater reductions in US-measured tumor size strongly correlated with achieving a T0 or Tis status on the pathology report. Additional predictors in the model included PR status (OR = 0.18, *p* < 0.001), indicating that PR-negative patients were more likely to experience significant regression, and HER-2/neu positivity (OR = 2.06, *p* = 0.017), highlighting the known responsiveness of HER-2-positive tumors to targeted therapies.

The Nagelkerke R^2^ of 0.410 indicates that the model explained a substantial portion of the variance in achieving significant regression, suggesting that US measurements combined with specific tumor characteristics (e.g., hormone receptor status) can be used to predict treatment outcomes with reasonable accuracy. The results are presented in Table 9.

These findings solidify the role of US as a non-invasive, practical, and effective tool for monitoring tumor response throughout chemotherapy. The significant correlation between US-derived percentage change and pathologic outcomes supports the use of ultrasound for real-time decision-making in clinical practice. By establishing that US can reliably approximate pT in identifying significant tumor regression, our study highlights the potential for integrating ultrasound more prominently into treatment protocols, thereby improving the ability of clinicians to assess tumor response, without relying solely on post-treatment pathology. This approach could lead to more dynamic treatment adjustments, improved patient management, and the optimization of therapeutic outcomes.

## 4. Discussion

Our study underscores the potential of US as a pivotal tool in monitoring tumor regression during neoadjuvant chemotherapy for breast cancer patients. Building upon our findings, it is imperative to delve deeper into the clinical implications, compare US with other imaging modalities, emphasize the need for standardization in sonographic measurements, explore the correlation between US-observed regression and patient outcomes, and outline future research directions. These findings align with prior research and contribute to the growing body of evidence supporting non-invasive, cost-effective alternatives for tracking chemotherapy response [9].

In our cohort, we observed a strong correlation between US-measured tumor regression and pathologic tumor response, reinforcing US as a valuable predictive tool. Notably, tumors demonstrating a >50% reduction in size by mid-treatment were significantly more likely to reach pathologic complete response (pCR) by the end of NAC. This finding is consistent with other studies that identified early tumor shrinkage on US as a key predictor of final treatment outcomes [17,18].

Moreover, our data indicate that HER2-positive tumors exhibited a more rapid size reduction on US compared to hormone receptor-positive/HER2-negative subtypes. This aligns with studies showing that HER2-targeted therapies induce early tumor regression, which is detectable through real-time US monitoring. Importantly, this suggests US could help tailor treatment strategies by distinguishing slow responders from rapid responders early in the treatment course [19,20,21].

The integration of US into routine clinical practice offers several advantages. Its real-time imaging capability allows clinicians to assess tumor response dynamically, facilitating timely modifications to treatment plans. For instance, early identification of non-responders through US could prompt a switch to alternative therapeutic strategies, potentially improving patient outcomes. Moreover, for patients exhibiting rapid tumor regression, US can aid in determining the feasibility of breast-conserving surgeries, thereby reducing surgical morbidity. The non-ionizing nature of US also permits frequent assessments without exposing patients to radiation, making it a patient-friendly option for ongoing monitoring. Studies have highlighted the utility of US in evaluating tumor vascularity changes during NAC, which can serve as early indicators of treatment efficacy [22]. Additionally, advancements in US technology, such as contrast-enhanced ultrasound (CEUS), have shown promise in enhancing the visualization of tumor perfusion and assessing therapeutic response [23].

While magnetic resonance imaging is often regarded as the gold standard for monitoring NAC response due to its high sensitivity and detailed tissue characterization, it is not without limitations. MRI can sometimes overestimate residual disease, leading to unnecessary surgical interventions [24]. In contrast, US offers a more accessible and cost-effective alternative. A prospective study comparing mammography, sonography, and MRI found that US had comparable accuracy in predicting residual tumor size post-NAC, with the added benefits of being more widely available and less expensive [6]. However, US is operator-dependent, and its accuracy can vary based on the technician’s experience and the equipment used. Combining US with other modalities, such as MRI or mammography, may enhance diagnostic accuracy and provide a more comprehensive assessment of tumor response [25].

The operator-dependent nature of US underscores the need for standardized imaging protocols to ensure consistency and reliability across different clinical settings. Standardization can minimize inter-operator variability and improve the reproducibility of measurements. The development of automated breast volume scanners (ABVSs) has been found to be more reliable than handheld US and as accurate as contrast-enhanced MRI in early detection of patients who could reach a pathological complete response after NAC [26]. Furthermore, the application of artificial intelligence (AI) and machine learning algorithms in US image analysis holds promise for reducing subjectivity and enhancing diagnostic accuracy. A study demonstrated that using dynamic US imaging changes acquired during NAC, combined with AI-based modeling, could predict pathological complete response noninvasively and early [27]. Implementing such technologies could lead to more objective assessments and better treatment planning.

Monitoring tumor response during NAC is crucial for predicting patient outcomes. Studies have shown that early changes in quantitative US imaging parameters can predict final treatment outcomes. For instance, a study demonstrated that using US image data, final outcomes could be predicted as early as within one month of starting chemotherapy [28]. This early prediction capability allows for timely adjustments in treatment strategies, potentially improving overall survival rates. Additionally, the ability of US to assess tumor vascularity and stiffness changes during therapy provides insights into tumor biology and treatment efficacy, further aiding in prognostication [29].

This is supported by Tran et al., who demonstrated that US-measured volume changes were significantly correlated with progression-free survival (PFS) and overall survival (OS) in NAC-treated breast cancer patients [30]. Furthermore, Hayashi et al. found that US-detected early shrinkage patterns were predictive of long-term recurrence risk, reinforcing its value in treatment stratification [31].

Beyond response monitoring, the pattern and timing of tumor shrinkage during neoadjuvant chemotherapy may have prognostic implications. Previous studies have shown that early volume reduction on imaging, including ultrasound, is associated with improved recurrence-free and overall survival [30,31]. In our cohort, we observed that tumors often underwent the most substantial reduction after four cycles, particularly in HER2-positive and hormone-receptor-positive groups. While survival data were not available in this study, the strong correlation between ultrasound-measured shrinkage and pathologic regression suggests that real-time ultrasound assessments may also serve as prognostic markers, helping identify patients at higher or lower risk based on their early response dynamics. Future prospective studies incorporating survival outcomes will be valuable in confirming these associations.

While our study highlights the potential of US in monitoring NAC response, further research is warranted to validate these findings across larger, diverse patient populations. Future studies should focus on multicenter trials to assess the generalizability of US as a monitoring tool. Additionally, exploring the integration of advanced US techniques, such as elastography and contrast-enhanced imaging, could provide more comprehensive assessments of tumor response. While this study utilized a consistent ultrasound device and standardized imaging protocol, formal intra- and inter-observer reproducibility analyses were not conducted and remain an important consideration for future prospective validations. Investigating the role of AI in automating US image analysis and developing predictive models for treatment outcomes is another promising avenue. Moreover, comparative studies evaluating the effectiveness of US against other imaging modalities in different clinical scenarios would help delineate the specific contexts in which US offers the most benefit. Addressing these areas will enhance the utility of US in clinical practice and contribute to more personalized treatment approaches for breast cancer patients undergoing NAC.

## 5. Conclusions

Our study supports the clinical utility of ultrasound as a dynamic, real-time tool for monitoring NAC response. The significant correlation between US-measured tumor regression and pathologic outcomes highlights its potential for guiding treatment decisions. US presents a cost-effective, accessible, and repeatable alternative, particularly in settings where frequent monitoring is required.

## Figures and Tables

**Table 1:** Categorical clinical and pathological characteristics of the study cohort.

Categorical Variable	Group	Count (%)
DCIS	No	265 (94%)
	Yes	17 (6%)
ER	Negative	119 (42.2%)
	Positive	163 (57.8%)
HER-2/neu	Negative	177 (62.8%)
	Positive	105 (37.2%)
PR	Negative	150 (53.2%)
	Positive	132 (46.8%)
TRG	0	5 (1.8%)
	1	96 (34%)
	2	54 (19.1%)
	3	11 (3.9%)
	4	116 (41.1%)
Affected Breast	L	152 (53.9%)
	R	130 (46.1%)
pT	T0	117 (41.5%)
	T1a	29 (10.3%)
	T1b	19 (6.7%)
	T1c	50 (17.7%)
	T2	50 (17.7%)
	T3	6 (2.1%)
	T4b	1 (0.4%)
	Tis	10 (3.5%)

Abbreviations: DCIS—ductal carcinoma in situ, ER—estrogen receptor, HER-2/neu—HER-2neu positivity, PR—progesterone receptor, TRG—tumor regression grade, pT—pathological tumor size.

**Table 2:** Distribution of continuous variables and results of normality testing.

Variable	Median (Q25-Q75)	*p*-Value
US-pre	27 (19–36)	<0.001
US-4C	16 (11–23)	<0.001
US-post	12 (8–18)	<0.001
Ki67	0.30 (0.20–0.50)	<0.001

Abbreviations: US-pre—ultrasound measurements before chemotherapy, US-4C—ultrasound measurements after 4-cycle chemotherapy, US-post—ultrasound measurements after chemotherapy, Ki67—Ki67 proliferation index, Q25-Q75—interquartile range, *p*-value—Shapiro–Wilk test result.

**Table 3:** Correlation between tumor regression grade (trg) and ultrasound measurements across all patients.

Variable	ρ (Rho)	*p*-Value
US-pre	−0.248	<0.001
US-4C	−0.448	<0.001
US-post	−0.547	<0.001
% Change Pre to Post	−0.488	<0.001

Abbreviations: US-pre—ultrasound measurements before chemotherapy, US-4C—ultrasound measurements after 4-cycle chemotherapy, US-post—ultrasound measurements after chemotherapy, ρ—Spearman’s rank correlation coefficient, *p*-value—Spearman’s rank correlation test result.

**Table 4:** Generalized estimating equations (GEE) model results for ultrasound-measured tumor size.

Predictors	Estimates	CI	*p*-Value
Time Point (US-4C)	−10.69	−11.86–−9.53	<0.001
Time Point (US-post)	−14.88	−16.19–−13.58	<0.001
TRG	−3.15	−3.98–−2.32	<0.001
HER-2/neu [+]	−3.09	−5.12–−1.05	0.003
McFadden R^2^ = 0.090			

Abbreviations: US-4C—ultrasound measurements after 4-cycle chemotherapy, US-post—ultrasound measurements after chemotherapy, TRG—tumor regression grade, CI—confidence interval, *p*-value—Wald test result.

**Table 5:** Median percentage tumor size reduction by hormone receptor and HER2 status.

Molecular Type		Median Reduction	IQR	*p*-Value
HER-2/neu	Positive	−50.0	34.6	0.048
	Negative	−58.3	27.9	
ER	Positive	−59.1	31.2	0.005
	Negative	−48.0	30.5	
PR	Positive	−58.0	32.3	0.004
	Negative	−47.3	29.9	

Abbreviations: HER-2/neu—HER-2/neu receptor positivity, ER—estrogen receptor, PR—progesterone receptor, IQR—inter quartile range, *p*-value—Kruskal–Wallis test result.

**Table 6:** Multinomial logistic regression model for ultrasound measurements before chemotherapy.

Predictors	Odds Ratios	CI	*p*-Value	Response
US-pre	1.11	1.01–1.22	0.031	T0
US-pre	1.12	1.02–1.24	0.020	T1a
US-pre	1.13	1.02–1.25	0.016	T1b
US-pre	1.12	1.01–1.23	0.024	T1c
US-pre	1.19	1.08–1.31	0.001	T2
US-pre	1.26	1.13–1.40	<0.001	T3
US-pre	1.18	1.01–1.38	0.040	T4b
AIC = 882.681				
BIC = 933.668				
Nagelkerke R^2^ = 0.187				

Abbreviations: US-pre—ultrasound measurements after chemotherapy, CI—confidence interval, *p*-value—Wald test result.

**Table 7:** Multinomial logistic regression model for ultrasound measurements after four cycles of chemotherapy.

Predictors	Odds Ratios	CI	*p*-Value	Response
US-4C	1.12	1.01–1.25	0.038	T1c
US-4C	1.26	1.12–1.41	<0.001	T2
US-4C	1.48	1.25–1.75	<0.001	T3
US-4C	1.35	1.09–1.68	0.006	T4b
AIC = 816.296				
BIC = 867.282				
Nagelkerke R^2^ = 0.366				

Abbreviations: US-4C—ultrasound measurements after 4 cycles of chemotherapy, CI—confidence interval, *p*-value—Wald test result.

**Table 8:** Multinomial logistic regression model for ultrasound measurements post-chemotherapy.

Predictors	Odds Ratios	CI	*p*-Value	Response
US-post	1.22	1.07–1.39	0.003	T1b
US-post	1.22	1.08–1.38	0.001	T1c
US-post	1.41	1.24–1.60	<0.001	T2
US-post	1.47	1.15–1.88	0.002	T4b
AIC = 751.46				
BIC = 802.133				
Nagelkerke R^2^ = 0.505				

Abbreviations: US-post—ultrasound measurements after chemotherapy, CI—confidence interval, *p*-value—Wald test result.

**Table 9:** Logistic regression analysis of significant tumor regression using ultrasound measurements.

Predictors	Odds Ratios	CI	*p*-Value
% Change Pre to Post	0.96	0.95–0.97	<0.001
PR [+]	0.18	0.10–0.32	<0.001
HER-2/neu [+]	2.06	1.15–3.74	0.017
AIC = 292.909			
BIC = 307.477			
R^2^ Nagelkerke = 0.410			

Abbreviations: PR—progesterone receptor status, CI—confidence interval, *p*-value—Wald test result.

## Data Availability

Further information concerning the present study is available from the corresponding author upon reasonable request.

## References

[1] American Cancer Society (2020). Global Cancer Facts & Figures.

[2] Varzaru V.B., Vlad T., Popescu R., Vlad C.S., Moatar A.E., Cobec I.M. (2024). Triple-Negative Breast Cancer: Molecular Particularities Still a Challenge. Diagnostics.

[3] Ochi T., Bianchini G., Ando M., Nozaki F., Kobayashi D., Criscitiello C., Curigliano G., Iwamoto T., Niikura N., Takei H. (2019). Predictive and prognostic value of stromal tumour-infiltrating lymphocytes before and after neoadjuvant therapy in triple negative and HER2-positive breast cancer. Eur. J. Cancer.

[4] Burciu O.M., Sas I., Popoiu T.A., Merce A.G., Moleriu L., Cobec I.M. (2024). Correlations of Imaging and Therapy in Breast Cancer Based on Molecular Patterns: An Important Issue in the Diagnosis of Breast Cancer. Int. J. Mol. Sci..

[5] Varzaru V.B., Eftenoiu A.E., Vlad D.C., Vlad C.S., Moatar A.E., Popescu R., Cobec I.M. (2024). The Influence of Tumor-Specific Markers in Breast Cancer on Other Blood Parameters. Life.

[6] Marinovich M.L., Macaskill P., Irwig L., Sardanelli F., von Minckwitz G., Mamounas E., Brennan M., Ciatto S., Houssami N. (2013). Meta-analysis of agreement between MRI and pathologic breast tumour size after neoadjuvant chemotherapy. Br. J. Cancer.

[7] Lobbes M.B.I., Jochelson M.S., Neeter L.M.F.H., Nelemans P.J. (2023). Contrast-enhanced Mammography and Breast MRI: Friends or Foes?. Radiology.

[8] McLaughlin R., Hylton N. (2011). MRI in breast cancer therapy monitoring. NMR Biomed..

[9] Piotrzkowska-Wróblewska H., Dobruch-Sobczak K., Klimonda Z., Karwat P., Roszkowska-Purska K., Gumowska M., Litniewski J. (2019). Monitoring breast cancer response to neoadjuvant chemotherapy with ultrasound signal statistics and integrated backscatter. PLoS ONE.

[10] Braman N., Adoui M.E., Vulchi M., Turk P., Etesami M., Fu P., Madabhushi A. (2020). Deep learning-based prediction of response to HER2-targeted neoadjuvant chemotherapy from pre-treatment dynamic breast MRI: A multi-institutional validation study. arXiv.

[11] Sharma D., Sannachi L., Osapoetra L.O., Cartar H., Cui W., Giles A., Czarnota G.J. (2024). Noninvasive Evaluation of Breast Tumor Response to Combined Ultrasound-Stimulated Microbubbles and Hyperthermia Therapy Using Quantitative Ultrasound-Based Texture Analysis Method. J. Ultrasound Med..

[12] Marinovich M.L., Sardanelli F., Ciatto S., Mamounas E., Brennan M., Macaskill P., Irwig L., von Minckwitz G., Houssami N. (2012). Early prediction of pathologic response to neoadjuvant therapy in breast cancer: Systematic review of the accuracy of MRI. Breast.

[13] Gentile D., Sagona A., De Carlo C., Fernandes B., Barbieri E., Di Maria Grimaldi S., Jacobs F., Vatteroni G., Scardina L., Biondi E. (2023). Pathologic response and residual tumor cellularity after neo-adjuvant chemotherapy predict prognosis in breast cancer patients. Breast.

[14] Liu L., Cui W.C., Sun Y., Wang H., Liang Z.N., Wu W., Yan K., Ji Y.L., Dong L., Yang W. (2025). Classification of Neoadjuvant Therapy Response in Patients With Colorectal Liver Metastases Using Contrast-Enhanced Ultrasound-With Histological Pathology as the Gold Standard. Ultrasound Med. Biol..

[15] Duan Y., Song X., Guan L., Wang W., Song B., Kang Y., Jia Y., Zhu Y., Nie F. (2023). Comparative study of pathological response evaluation systems after neoadjuvant chemotherapy for breast cancer: Developing predictive models of multimodal ultrasound features including shear wave elastography combined with puncture pathology. Quant. Imaging Med. Surg..

[16] Eisenhauer E.A., Therasse P., Bogaerts J., Schwartz L.H., Sargent D., Ford R., Dancey J., Arbuck S., Gwyther S., Mooney M. (2009). New response evaluation criteria in solid tumours: Revised RECIST guideline (version 1.1). Eur. J. Cancer.

[17] Eun N.L., Son E.J., Gweon H.M., Kim J.A., Youk J.H. (2020). Prediction of axillary response by monitoring with ultrasound and MRI during and after neoadjuvant chemotherapy in breast cancer patients. Eur. Radiol..

[18] Rix A., Piepenbrock M., Flege B., von Stillfried S., Koczera P., Opacic T., Simons N., Boor P., Thoröe-Boveleth S., Deckers R. (2021). Effects of contrast-enhanced ultrasound treatment on neoadjuvant chemotherapy in breast cancer. Theranostics.

[19] Liu Y., Wang Y., Wang Y., Xie Y., Cui Y., Feng S., Yao M., Qiu B., Shen W., Chen D. (2022). Early prediction of treatment response to neoadjuvant chemotherapy based on longitudinal ultrasound images of HER2-positive breast cancer patients by Siamese multi-task network: A multicentre, retrospective cohort study. EClinicalMedicine.

[20] Wang X., Zhang Y., Yang M., Wu N., Wang S., Chen H., Zhou T., Zhang Y., Wang X., Jin Z. (2024). Dynamic ultrasound-based modeling predictive of response to neoadjuvant chemotherapy in patients with early breast cancer. Sci. Rep..

[21] Sui L., Yan Y., Jiang T., Ou D., Chen C., Lai M., Ni C., Zhu X., Wang L., Yang C. (2023). Ultrasound and clinicopathological characteristics-based model for prediction of pathologic response to neoadjuvant chemotherapy in HER2-positive breast cancer: A case-control study. Breast Cancer Res. Treat..

[22] Dobruch-Sobczak K., Piotrzkowska-Wróblewska H., Klimoda Z., Secomski W., Karwat P., Markiewicz-Grodzicka E., Kolasińska-Ćwikła A., Roszkowska-Purska K., Litniewski J. (2019). Monitoring the response to neoadjuvant chemotherapy in patients with breast cancer using ultrasound scattering coefficient: A preliminary report. J. Ultrason..

[23] Adrada B.E., Candelaria R., Moulder S., Thompson A., Wei P., Whitman G.J., Valero V., Litton J.K., Santiago L., Scoggins M.E. (2021). Early ultrasound evaluation identifies excellent responders to neoadjuvant systemic therapy among patients with triple-negative breast cancer. Cancer.

[24] Chen J.H., Feig B.A., Hsiang D.J., Butler J.A., Mehta R.S., Bahri S., Nalcioglu O., Su M.Y. (2009). Impact of MRI-evaluated neoadjuvant chemotherapy response on change of surgical recommendation in breast cancer. Ann. Surg..

[25] Peintinger F., Kuerer H.M., Anderson K., Boughey J.C., Meric-Bernstam F., Singletary S.E., Hunt K.K., Whitman G.J., Stephens T., Buzdar A.U. (2006). Accuracy of the combination of mammography and sonography in predicting tumor response in breast cancer patients after neoadjuvant chemotherapy. Ann. Surg. Oncol..

[26] Liu X., Dai Y., Wu Y., Li F., Liang M., Wu Q. (2025). Diagnostic accuracy of automated breast volume scanning, hand-held ultrasound and molybdenum-target mammography for breast lesions: A systematic review and meta-analysis. Gland. Surg..

[27] Tadayyon H., Sannachi L., Gangeh M., Sadeghi-Naini A., Tran W., Trudeau M.E., Pritchard K., Ghandi S., Verma S., Czarnota G.J. (2016). Quantitative ultrasound assessment of breast tumor response to chemotherapy using a multi-parameter approach. Oncotarget.

[28] Guo J., Wang B.H., He M., Fu P., Yao M., Jiang T. (2022). Contrast-enhanced ultrasonography for early prediction of response of neoadjuvant chemotherapy in breast cancer. Front. Oncol..

[29] Wu L., Ye W., Liu Y., Chen D., Wang Y., Cui Y., Li Z., Li P., Li Z., Liu Z. (2022). An integrated deep learning model for the prediction of pathological complete response to neoadjuvant chemotherapy with serial ultrasonography in breast cancer patients: A multicentre, retrospective study. Breast Cancer Res..

[30] Tran W.T., Childs C., Chin L., Slodkowska E., Sannachi L., Tadayyon H., Watkins E., Wong S.L., Curpen B., El Kaffas A. (2016). Multiparametric monitoring of chemotherapy treatment response in locally advanced breast cancer using quantitative ultrasound and diffuse optical spectroscopy. Oncotarget.

[31] Hayashi M., Yamamoto Y., Iwase H. (2020). Clinical imaging for the prediction of neoadjuvant chemotherapy response in breast cancer. Chin. Clin. Oncol..

